# Automated quality control in nuclear medicine using the structured noise index

**DOI:** 10.1002/acm2.12850

**Published:** 2020-04-11

**Authors:** Jeffrey S. Nelson, Ehsan Samei

**Affiliations:** ^1^ Clinical Imaging Physics Group Department of Radiology Duke University Medical Center Durham NC USA; ^2^ Carl E. Ravin Advanced Imaging Laboratories Medical Physics Graduate Program Departments of Radiology Biomedical Engineering, and Electrical and Computer Engineering Duke University Durham NC USA

**Keywords:** automated analysis, Gamma camera, quality assurance, uniformity

## Abstract

**Purpose:**

Daily flood‐field uniformity evaluation serves as the central element of nuclear medicine (NM) quality control (QC) programs. Uniformity images are traditionally analyzed using pixel value‐based metrics, that is, integral uniformity (IU), which often fail to capture subtle structure and patterns caused by changes in gamma camera performance, requiring visual inspections which are subjective and time demanding. The goal of this project was to implement an advanced QC metrology for NM to effectively identify nonuniformity issues, and report issues in a timely manner for efficient correction prior to clinical use. The project involved the implementation of the program over a 2‐year period at a multisite major medical institution.

**Methods:**

Using a previously developed quantitative uniformity analysis metric, the structured noise index (SNI) [Nelson et al. (2014), \textit{J Nucl Med.}, \textbf{55}:169—174], an automated QC process was developed to analyze, archive, and report on daily NM QC uniformity images. Clinical implementation of the newly developed program ran in parallel with the manufacturer’s reported IU‐based QC program. The effectiveness of the SNI program was evaluated over a 21‐month period using sensitivity and coefficient of variation statistics.

**Results:**

A total of 7365 uniformity QC images were analyzed. Lower level SNI alerts were generated in 12.5% of images and upper level alerts in 1.7%. Intervention due to image quality issues occurred on 26 instances; the SNI metric identified 24, while the IU metric identified eight. The SNI metric reported five upper level alerts where no clinical engineering intervention was deemed necessary.

**Conclusion:**

An SNI‐based QC program provides a robust quantification of the performance of gamma camera uniformity. It operates seamlessly across a fleet of multiple camera models and, additionally, provides effective workflow among the clinical staff. The reliability of this process could eliminate the need for visual inspection of each image, saving valuable time, while enabling quantitative analysis of inter‐ and intrasystem performance.

## 1 INTRODUCTION

In nuclear medicine (NM), a robust quality control (QC) program is essential for detecting detrimental changes in camera performance, allowing for remediation prior to clinical involvement.^1^, ^2^, ^3^ Currently, one of the most valuable assessments is the uniformity evaluation, routinely performed on a daily basis prior to patient imaging. Many of the most significant performance issues will produce system nonuniformities which are effectively depicted in the uniformity evaluation image. Early identification of performance issues provides the operator a chance to initiate appropriate corrective action prior to patient imaging, preventing suboptimal clinical studies. However, in order to benefit from this valuable quality control evaluation, a suitable and reliable process must be in place.

Typically the traditional uniformity evaluation process involves image acquisition, followed by analysis comprised of a critical visual inspection, in addition to pixel value‐based analysis.^4^, ^5^, ^6^ While a critical visual inspection is considered the gold standard and should be performed prior to pixel‐based analysis,^7^ several downfalls exist. First, it is time consuming, especially during the busy morning when the technologist is preparing for patients, potentially resulting in an insufficient inspection. In addition, it is also subjective; relying heavily on the expertise of the reviewer to determine which nonuniformities may have a clinical impact. In order to make the evaluation process more objective and to provide an additional perspective on image uniformity, a computer analysis program typically accompanies the visual analysis. However, the traditional pixel value‐based programs often fail to adequately identify subtle structure and patterns, potentially biasing the reviewer to perhaps erroneously pass an image with visual nonuniformities.

In a previous project, a new uniformity analysis metric was developed, the structured noise index (SNI), which reports the uniformity of an image based on the image noise texture.^8^ It was found that this metric outperforms currently established pixel value‐based analysis methods for identifying image nonuniformities and additionally, correlates closely with expert visual analysis possibly reducing the need for visual assessment.

In this current project, we evaluate the practicality of integrating the SNI metric into the daily uniformity QC program, where oftentimes a qualified medical physicist is unable to visually analyze the quality of each flood image prior to patient studies. The goal of this project was to develop and implement a robust QC metrology for NM that is effective and reliable in identifying nonuniformity issues, effective in reporting issues in a timely manner for effective problem correction, and to characterize the program over a 2‐yr period in an academic medical center setting.

## 2 MATERIALS AND METHODS

### 2.1 Structured noise index

The SNI was developed in a previous project for quantifying nuclear medicine flood‐field uniformity images with regard to the presence of nonuniformities, detailed in an earlier publication.^8^ In summary, the SNI is based on frequency‐based two‐dimensional (2D) noise power spectrum (NPS),

(1) $$NPS\left(u_{n},v_{k}\right)=\lim_{N_{x},N_{y},M\to \infty }\frac{\Delta_{x}\Delta_{y}}{M\cdot N_{x}N_{y}}\sum_{m=1}^{M}{\left|\sum_{u=1}^{N_{x}}\sum_{j=1}^{N_{y}}\left[I\left(x_{i},y_{j}\right)-\bar{I}\right]e^{-2\pi i\left(u_{n}x_{i}+v_{k}y_{i}\right)}\right|}^{2}$$

where $I\left(x_{i},y_{j}\right)$ is the image intensity at pixel location $\left(x_{i},y_{j}\right)$, $\bar{I}$ is the global mean intensity, u and v are the spatial frequencies conjugate to x and y, $N_{x}$ and $N_{y}$ are the number of pixels in the x and y directions, $\Delta_{x}$ and $\Delta_{y}$ are the pixel spacing in the x and y directions, and M is the number of regions used for analysis in the ensemble average which can be adequately reported with a sufficiently large number of regions.

The input flood SNI value is computed by subtracting the estimated quantum component of the flood image (based on the variance associated with the number of counts in the image) from the generated two‐dimensional (2D) NPS of the input flood image, resulting in a 2D NPS of only the structured noise within the image. Both the input NPS and structured noise NPS are further filtered with a 2D human visual response function using the equation

(2) $$V\left(r\right)=r^{1.3}\cdot \exp\left(-cr^{2}\right)$$

where *r* is the radial spatial frequency and *c* is a scale factor selected to yield the maximum for the function at four cycles per degree at a typical viewing distance of 150 cm and typical image display size of 6.5 cm. The input flood SNI value is the ratio of the filtered NPS of the structured noise to the filtered NPS of the input image as given by the following equation

(3) $$\frac{\int NPS_{structure,filtered}\;dudv}{\int NPS_{input,filtered}\;dudv}.$$

The SNI metric was validated through an observer study, and further compared against some traditional pixel value‐based uniformity analysis metrics. The SNI outperformed the traditional metrics in both identifying nonuniformities and correlating with expert visual analysis.

For this project slight modifications were made to the regions of interest (ROIs) used for SNI analysis. From the original project, the two large equally sized overlapping square ROIs encompassing the central 90% of the full image remain unchanged.^8^ However, the six additional ROIs were replaced with a small roving 64 × 64 pixel ROI (about 25% of the height of a typical flood image acquired in 256 × 256 matrix) to better detect local defects, for example, failing photomultiplier tubes. Analysis is performed with the small ROI beginning in the upper left corner and using shifting increments of 16 pixels at a time until the ROI has swept over the entire image (approximately 150 total ROI's for a typical flood image). The ROI returning the highest SNI score is assigned as the image SNI score.

Additionally, an Artifact Image is created during the SNI analysis, which is useful for visually identifying nonuniformities. This Artifact Image is generated by applying a threshold in the Fourier domain of the image to isolate the components that are outside the expected values assuming Gaussian noise. An inverse Fourier transform is then applied to generate the Artifact Image.

### 2.2 Automated analysis process

To effectively put the SNI analysis into clinical use, an automated process was developed to transmit, receive, analyze, report, and archive the daily uniformity images.

#### 2.2.1 Transmission

A physics network database was first created within the medical center network. This database was then entered as a “send” destination on each NM imaging system. After acquisition, each system sends the uniformity image to the physics database in DICOM format where they are then sorted based on physical and acquisition attributes contained in the DICOM header (i.e., station name, study type, number of counts, etc.). This sorting process helps avoid images which are not uniformity QC images from being analyzed by the program.

#### 2.2.2 Analysis

**Fig. 1:**
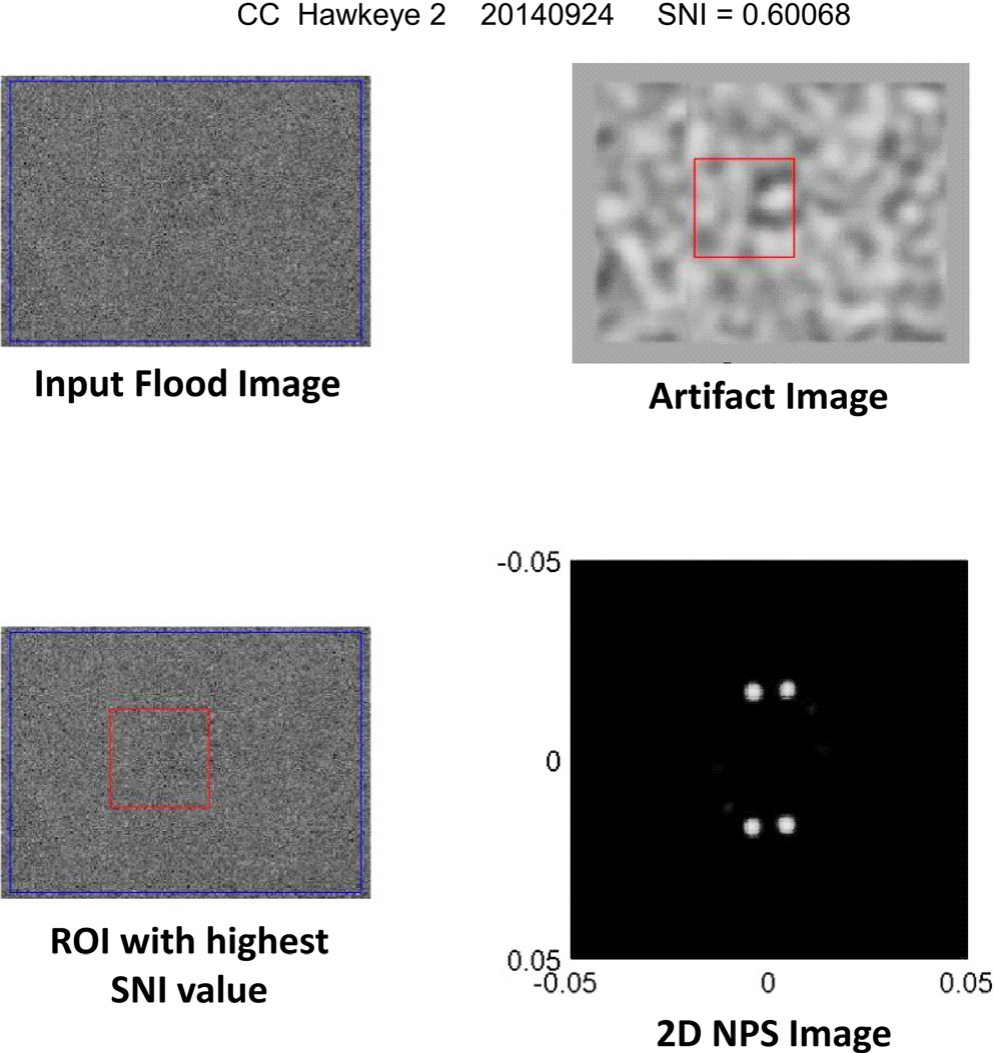
Output figure generated during structured noise index (SNI) analysis. In this example, the SNI program accurately identified a non‐uniformity visually apparent in the center of the input image.

After the received images are properly sorted, the uniformity images are then analyzed using the SNI metric described above. As part of the SNI analysis, a four‐quadrant figure shown in Fig. 1 is also generated which provides the user with a visual depiction of any identified nonuniformities. The figure contains four images; the upper left quadrant contains the input uniformity image, lower left the ROI reporting the highest SNI value, lower right the 2D NPS of the highest ROI, and upper right the Artifact Image.

#### 2.2.3 Reporting

Results of the SNI analysis as well as image acquisition attributes (DICOM header information) are populated into a database assessable by both physics and clinical staff. Alert thresholds are defined for SNI values; if the SNI value exceeds the defined threshold value, an email notification with a jpeg attachment of the image, is immediately sent to the defined recipients initiating prompt investigation of the potential nonuniformity. At our institution, we set two threshold alert levels based on the expert observer responses of our 55 test images from our previous publication. An upper‐level threshold, triggering email notification for physics, clinical, and clinical engineering staff was set at 0.60, which corresponds to a sensitivity of 61.5% and a positive predictive value of 100%. A lower level threshold, triggering email notification for physics staff only was set at 0.50, which increases the sensitivity to 100% while lowering the positive predictive value to 86.7%. A user can set a low threshold to ensure maximum sensitivity, however, this will likely increase false positive alerts requiring further detailed visual inspection of likely acceptable images.

#### 2.2.4 Archiving

After the uniformity images are analyzed, they are automatically archived in separate folders based on acquisition station name. These archived images provide the physicist the ability to view and further analyze the images via remote access. The four‐quadrant jpeg images are also stored in separate folders based on station name. Archival allows interrogation of the recent history of a detector for gradual developments.

### 2.3 Clinical validation

**Fig. 2:**
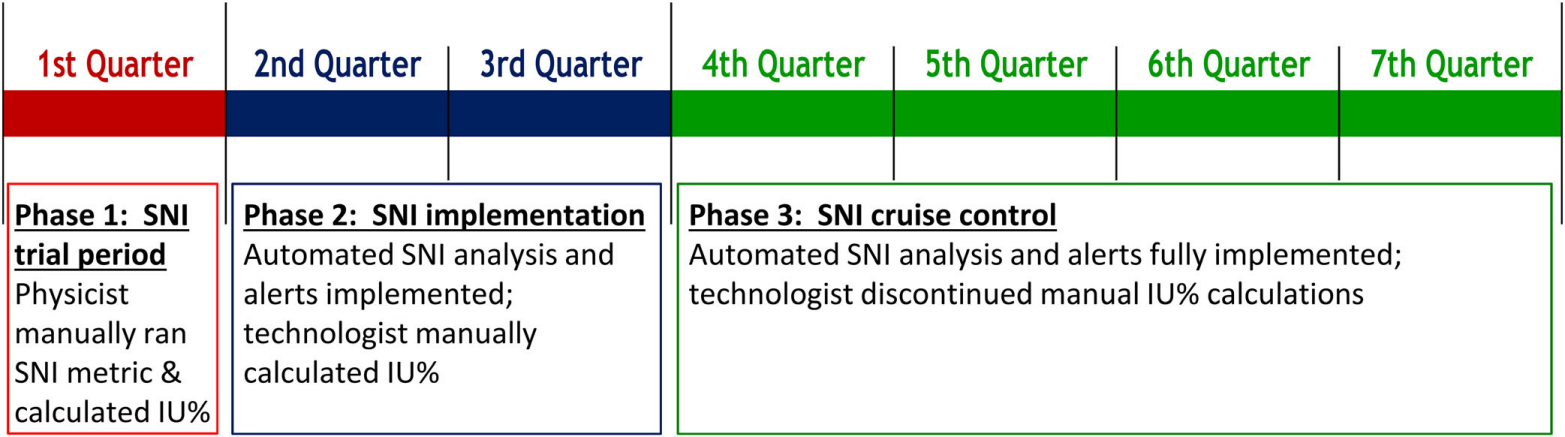
The three phases of the structured noise index clinical validation.

The utility of the program was tested for a period of 21 months at an academic medical center utilizing nine nuclear imaging systems (Millennium MyoSIGHT, Millennium MPR, Infinia series [× 3], and Discovery series [× 4], all from GE Healthcare) at three different locations. The trial was divided into three phases which are shown in Fig. 2. The first phase covered 3 months during which the physics staff traveled to the nuclear medicine department each morning to perform a through visual inspection of each uniformity image from every system. The images were then manually transferred to a removable storage device, loaded onto a physics computer, and manually run locally through the SNI program. Results were then manually entered into a spreadsheet. Additionally, the uniformity images were manually run through the GE Xeleris Uniformity Analysis (UA) program^9^ (per department protocol) and the integral uniformity (IU) values from the useful field of view (center 90% of the full field) and central field of view (center 75% of useful field of view linear dimensions) were also entered into the spreadsheet. During this first phase the nuclear medicine technologists continued their established protocol of performing a visual inspection, manually running the uniformity image through GE’s UA program, and manually recording results in the NM QC binder.

The second phase of the clinical validation covered the subsequent 6 months. Similar to the first phase, the physics staff physically traveled to the nuclear medicine department and performed a through visual inspection of each image. However, for this second phase, the SNI program was loaded onto the physics server, and one of the NM processing stations was linked to this server allowing manual transfer of the image file directly to the SNI program. After the images were manually transferred to the program, the images were analyzed and results were automatically populated into a spreadsheet. Additionally, for comparison purposes, the physics staff recorded the system‐generated uniformity percentage results from the NM Daily QC program incorporated in the Infinia^10^ and Discovery^11^ series systems. During the second phase the clinical staff continued visual inspection of each image, manually analyzing each image using GE’s UA analysis program, and entering results into the NM QC binder.

The third phase of the clinical validation covered the next 12 months. During this phase, the SNI program was further enhanced allowing direct image transfer between the NM system and the SNI program after the completion of the acquisition. The uniformity QC images were either manually transferred to the physics server by the technologist, or, where available, automatically transferred by the system at the conclusion of the QC acquisition. The SNI program was also further upgraded to provide email alerts if the SNI value exceeded a user defined threshold or if daily QC images were not received. Similar to the first two phases, the NM technologists continued to visually inspect each uniformity image, but discontinued manual analysis using the GE UA program. However, they did record the system‐generated uniformity percentage reported during the GE NM Daily QC Procedure.

### 2.4 Statistical analysis

The performance of the automated SNI uniformity analysis QC program was evaluated quarterly by calculating the sensitivity. A true positive was defined as an instance when the SNI metric returned a value exceeding the upper‐level threshold and service was performed on the system due to image quality issues. Instances when the threshold was exceeded and it was determined servicing was not necessary is a false positive. The sensitivity using the IU metric (generated from the GE UA program, or GE NM Daily QC program) was also calculated and reported for comparison purposes.

Variation in flood image quality was also evaluated using the coefficient of variation (CV) of the SNI which was calculated for each system on a quarterly basis. An overall average CV for each quarter was then calculated by dividing the average standard deviation of all systems by the average mean of all systems during the entire quarter.

## 3 RESULTS

A total of 7365 daily uniformity QC images were analyzed during the 21‐month trial. The automated SNI program generated lower level alerts in 12.5% of images and upper level alerts in 1.7% of images compared with 0.5% of images exceeding the alert level using the IU metrics. Clinical engineering intervention due to image quality issues occurred on 26 instances. Note there were some instances where the repair lasted several days, resulting in multiple poor quality flood images for the same issue. From the 26 instances, 24 were correctly identified by the SNI metric. The SNI also reported five upper level alerts where no clinical engineering intervention occurred. Compared to that, the IU correctly identified 8 of the 26 instances, and reported one false positive alert where no engineering intervention was warranted.

**Fig. 3:**
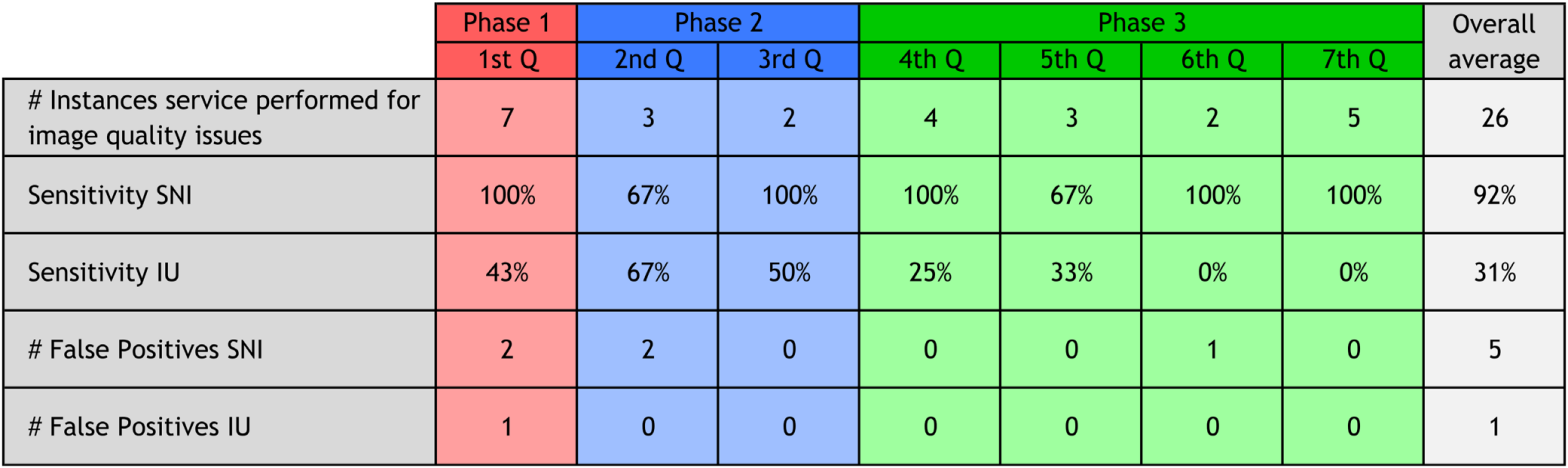
Sensitivity and false positive results during the three phases of the structured noise index clinical validation.

The SNI proved to be a better predictor of image quality issues than the IU metric determined by the sensitivity in identifying image quality issues at consistently low false positive rates. During the first phase of our SNI trial, the SNI successfully identified 100% of the seven image quality issues, while the IU metric identified only 43% of the issues. During the entire 21 months of our trial, the overall average SNI sensitivity was 92%, compared with 31% for the IU metric. The complete results of all three phases delineated by quarter are reported in Fig. 3.

**Fig. 4:**
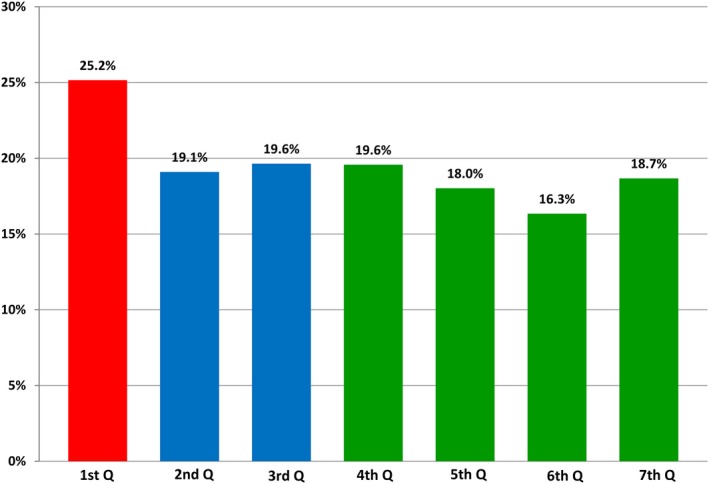
Average coefficient of variation across all systems throughout implementation of the structured noise index program.

In addition to the improved sensitivity, the average coefficient of variation also improved during each phase of implementation of the SNI program. During the first phase of implementation, the average CV of the SNI deceased by almost 25% and showed further improvement during the third phase indicating the day‐to‐day flood image quality across our system became more consistent throughout the duration of our implementation. The detailed results for each quarter are reported in Fig. 4.

## 4 DISCUSSION

Clinical implementation of the automated SNI program for daily uniformity QC analysis delivers improved detection sensitivity to clinically relevant uniformity artifacts with little or no added effort by the nuclear medicine technologists. In place of processing the image through the vendor provided analysis program, the technologist simply transfers the file to the SNI program and results are available within a matter of minutes. If the technologist forgets to look at the results of the image, there is additional security that all failing images will trigger email notification sent to several key individuals at the institution. To ensure the uniformity of all systems is assessed prior to use, the program is equipped with a feature to send email alerts if images are not received from a system. Additionally, the SNI program may potentially avoid personnel from needlessly spending time analyzing images with superior uniform quality.

Utilization of the program has also expedited the communication between clinical staff, clinical engineering, and physics when a problem occurs. Acquisition of the uniformity image typically takes place very early in the morning by the third or first shift technologist. If a system fails the uniformity analysis, the email alert will be sent immediately to the medical physicist who can make a decision if the system should be used clinically, and to the clinical engineer who may be able to troubleshoot the problem before arriving on site.

**Fig. 5:**
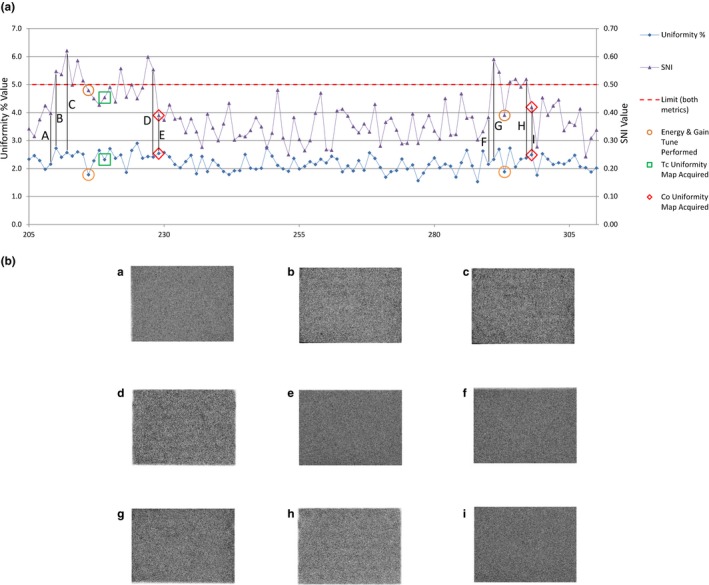
(a) Structured noise index (SNI) and system generated Uniformity % for a single detector over a 6 month period. The red horizontal line indicates the threshold used for both metrics (5.0% for Uniformity % and 0.50 for SNI). Instances where service was performed due to visual image quality issues are represented by hollow markers. This particular system uses isotope specific uniformity correction maps. When a Tc uniformity map was acquired, the clinical image (using Tc‐99m isotopes) quality became acceptable. However, the quality control (using Co‐57) continued to exceed limits until the Co uniformity correction was able to be acquired. (b) Flood images corresponding to locations indicated in (a) with enhanced window/level settings to better visualize non‐uniformities.

Figure 3 demonstrates the improved sensitivity in identifying images with clinically relevant artifacts the SNI program was able to provide. Results of a single detector over a 6‐month period, shown in Fig. 5(a), show SNI along with the system‐generated Uniformity % reported from the GE NM Daily QC Program. Elevated SNI values coincide with the two instances our institution determined service was needed to remedy image quality issues, however, the Uniformity % remained relatively unchanged and did not exceed the manufacturer’s acceptable range of <5.0%. Meanwhile, the SNI value spiked during the periods, exceeding our facility’s notification threshold of 0.50. The flood images at nine instances during the 6‐month period are included in Fig. 5(b).

Although the majority of clinical imaging is performed with Tc‐99m radiopharmaceuticals, traditionally daily quality control is performed using the more convenient Co‐57 sealed sheet source. Although the two are similar in energy (140 vs 122 keV), some camera systems use separate uniformity correction files for each isotope. Under these circumstances, one may encounter instances where the Co‐57 uniformity evaluated during quality control testing contains a nonuniformity, while the Tc‐99m uniformity may be uniform, or vice versa. In the example presented in Fig. 5, the camera system used isotope specific uniformity correction maps. For this reason, instances occurred where the Co‐57 QC results exceeds trigger thresholds for several days in a row before the new Co‐57 correction map is updated; however, the clinically relevant Tc‐99m uniformity was evaluated and repaired on the first day of notification. This is one example where the same clinical engineering intervention resulted in several upper‐level threshold alerts.

During the 21‐month SNI trial, two instances occurred when image quality‐related service was performed without an SNI‐triggered alert. In both instances, the SNI code did identify nonuniform texture within the image in the area deemed unacceptable by visual inspection, however, the SNI value did not exceed the facility established trigger threshold. One may consider adjusting the trigger threshold to be more sensitive to image nonuniformities, however, this adjustment will lead to an increase in the false positive rate.

Our evaluation of the SNI metric in a clinical quality control setting was based on comparison with a pixel value‐based metric (IU%). Although our evaluation demonstrates instances where monitoring the SNI metric proved to be a better predictor of uniformity, it should be mentioned that monitoring the IU% or other QC strategies (e.g., energy peak or energy resolution) will likely complement each other in order to provide a more complete assessment of quality in the nuclear medicine operation. This topic merits further investigation, but is beyond the scope of this paper.

## 5 CONCLUSION

Visual evaluation of NM daily uniformity QC images is time consuming, subjective, and prone to transcription and oversight (incomplete visual inspection) errors. Alternatively, the SNI provides a robust quantification of the NM performance of gamma camera uniformity in a more objective and quantitative fashion. Implementing across a large academic institution, it operates seamlessly across a fleet of multiple camera models. The automated alert process provides enhanced workflow between physicists, technologists, and clinical engineers. The reliability of this process paired with the high sensitivity of the SNI has made it the preferred platform for NM uniformity analysis, and could eliminate the need for visual inspection of each image.

## CONFLICT OF INTEREST

No conflict of interest.

## References

[1] Hines H , Kayayan R , Colsher J , et al. National Electrical Manufacturers Association recommendations for implementing SPECT instrumentation quality control. J Nucl Med. 2000;41:383–389. 10688125

[2] Smith EM . Scintillation camera quality control, part I: establishing the quality control program. J Nucl Med Technol. 1998;26:9–13. 9549686

[3] Busemann Sokole E , Plachcinska A , Britten A , et al. Routine quality control recommendations for nuclear medicine instrumentation. Eur J Nucl Med Mol Imaging. 2010;37:662–671. 20130859 10.1007/s00259-009-1347-y

[4] National Electrical Manufacturers Association . Performance Measurements of Gamma Cameras. Rosslyn, VA: National Electircal Manufacturers Association; 2007.

[5] American Association of Physicists in Medicine. Nuclear Medicine Committee . Scintillation Camera Acceptance Testing and Performance Evaluation. New York, NY: American Institute of Physics; 1980.

[6] Murray AW , Barnfield MC , Thorley PJ . Optimal uniformity index selection and acquisition counts for daily gamma camera quality control. Nucl Med Commun. 2014;35:1011–1017. 25029245 10.1097/MNM.0000000000000167

[7] American Association of Physicists in Medicine. Nuclear Medicine Committee . Computer‐Aided Scintillation Camera Acceptance Testing. New York, NY: American Institute of Physics; 1982.

[8] Nelson JS , Christianson OI , Harkness BA , et al. Improved nuclear medicine uniformity assessment with noise texture analysis. J Nucl Med. 2014;55:169–174. 24212975 10.2967/jnumed.113.125450

[9] GE Medical Systems . Flood Field Uniformity Protocol for Xeleris Functional Imaging P&R Systems: Operator Guide. Milwaukee, WI: General Electric Company; 2003.

[10] General Electric Medical Systems . Infinia User's Guide Nuclear Medicine Imaging System. Tirat Hacarmel, Israel: GE Medical Systems Israel; 2005.

[11] GE Healthcare . Discovery NM/CT 670 Quality Control Reference Manual. Milwaukee, WI: General Electric Company; 2010.

